# Identification of a cytokine-dominated immunosuppressive class in squamous cell lung carcinoma with implications for immunotherapy resistance

**DOI:** 10.1186/s13073-022-01079-x

**Published:** 2022-07-08

**Authors:** Minglei Yang, Chenghao Lin, Yanni Wang, Kang Chen, Haiyue Zhang, Weizhong Li

**Affiliations:** 1 Zhongshan School of Medicine, Sun Yat-sen University, Guangzhou, 510080 China; 2 Center for Precision Medicine, Sun Yat-sen University, Guangzhou, 510080 China; 3 Key Laboratory of Tropical Disease Control of Ministry of Education, Sun Yat-Sen University, Guangzhou, 510080 China

**Keywords:** Immunogenomics, LUSC, T cell exhaustion, Immunosuppressive cytokine, Immune checkpoint blockade resistance, Tumour microenvironment

## Abstract

**Background:**

Immune checkpoint blockade (ICB) therapy has revolutionized the treatment of lung squamous cell carcinoma (LUSC). However, a significant proportion of patients with high tumour PD-L1 expression remain resistant to immune checkpoint inhibitors. To understand the underlying resistance mechanisms, characterization of the immunosuppressive tumour microenvironment and identification of biomarkers to predict resistance in patients are urgently needed.

**Methods:**

Our study retrospectively analysed RNA sequencing data of 624 LUSC samples. We analysed gene expression patterns from tumour microenvironment by unsupervised clustering. We correlated the expression patterns with a set of T cell exhaustion signatures, immunosuppressive cells, clinical characteristics, and immunotherapeutic responses. Internal and external testing datasets were used to validate the presence of exhausted immune status.

**Results:**

Approximately 28 to 36% of LUSC patients were found to exhibit significant enrichments of T cell exhaustion signatures, high fraction of immunosuppressive cells (M2 macrophage and CD4 Treg), co-upregulation of 9 inhibitory checkpoints (*CTLA4*, *PDCD1*, *LAG3*, *BTLA*, *TIGIT*, *HAVCR2*, *IDO1*, *SIGLEC7*, and *VISTA*), and enhanced expression of anti-inflammatory cytokines (e.g. TGFβ and CCL18). We defined this immunosuppressive group of patients as exhausted immune class (EIC). Although EIC showed a high density of tumour-infiltrating lymphocytes, these were associated with poor prognosis. EIC had relatively elevated PD-L1 expression, but showed potential resistance to ICB therapy. The signature of 167 genes for EIC prediction was significantly enriched in melanoma patients with ICB therapy resistance. EIC was characterized by a lower chromosomal alteration burden and a unique methylation pattern. We developed a web application (http://lilab2.sysu.edu.cn/tex & http://liwzlab.cn/tex) for researchers to further investigate potential association of ICB resistance based on our multi-omics analysis data.

**Conclusions:**

We introduced a novel LUSC immunosuppressive class which expressed high PD-L1 but showed potential resistance to ICB therapy. This comprehensive characterization of immunosuppressive tumour microenvironment in LUSC provided new insights for further exploration of resistance mechanisms and optimization of immunotherapy strategies.

**Supplementary Information:**

The online version contains supplementary material available at 10.1186/s13073-022-01079-x.

## Background

Lung cancer is the most common cancer and the leading cause of cancer-related death worldwide [1]. Non-small-cell lung cancer (NSCLC) accounts for approximately 85% of lung cancer cases [2]. Besides lung adenocarcinoma, lung squamous carcinoma (LUSC) is the most frequent histologic subtype of NSCLC [3]. However, due to the lack of genetic alterations for which targeted treatments are approved, the patients of LUSC have limited treatment options except chemotherapy [4].

With the recent development of immune checkpoint blockade (ICB) immunotherapy, anti-PD-1/PD-L1 immune checkpoint inhibitors have been approved for the first-line treatment of NSCLC in patients with high tumour programmed death-ligand 1 (PD-L1) expression (≥50%) [5]. However, only 45.2% of patients met the screening criteria and benefited from the immune checkpoint blockade therapy [6, 7]. A significant fraction of patients had drug resistance to this immunotherapy. On the other hand, ICB therapy can cause unique toxicity and subsequently immune-related adverse events through enhanced immune responses [8].

ICB therapy aims to reinvigorate dysfunctional or exhausted T cells to eliminate tumours [9]. Tumour microenvironment (TME) consists of cancer cells, immune cells, stromal cells, and cytokines. The TME components dynamically regulated T cell exhaustion [10]. The success of ICB therapy by reinvigorating exhausted T cell relies heavily on the complex interactions between the cancer cells and the components of TME [11]. However, the knowledge of molecular mechanism of resistance to immune checkpoint inhibitors is limited. Therefore, to predict the patients’ response or resistance to ICB therapy and to tailor reasonable treatments, characterizing TME molecular features and consequently identifying potential therapeutic markers are greatly required.

The TME of LUSC is highly complex and heterogeneous, but little is known about how TME impacts the efficacy of immunotherapy in LUSC. Virtual microdissection analytical approach based on non-negative matrix factorization (NMF) has enabled effective deconvolution on the gene expression signals derived from tumour cells, inflammatory cells, stromal cells, and cytokines from bulk tumour samples [12, 13]. In this study, we aimed to dissect the RNAseq expression data of 624 human LUSC samples and isolate transcriptomic signals released from immunosuppressive TME through NMF. Consequently, we identified and validated an exhausted immune class of LUSC with immunosuppressive molecular features and potential ICB resistance. We also associated the exhausted immune class with multi-omics data to investigate the underlying ICB resistance mechanism. Finally, we constructed an interactive web application for researcher to explore immunotherapy resistance based on the multi-omics analysis results.

## Methods

### LUSC datasets and resources

The gene expression profiles of total 624 LUSC human samples were retrieved from The Cancer Genome Atlas (TCGA) (497 bulk RNA sequencing [RNAseq] datasets) [14] and Gene Expression Omnibus (GEO) (127 microarray datasets) (Additional file 1: Fig. S1). Due to the differences of clinical features and treatment regimens resulted from different tumour stages, we divided the 497 TCGA LUSC patients into the late-stage group (stage IIA to IV, 250 patients) for training and the early-stage group (stage I to II, 247 patients) for internal validation. The relevant data of mutation, copy number variation, methylation, and clinic pathology were obtained from the TCGA Data Portal (https://tcga-data.nci.nih.gov/tcga/, June 4th, 2020). The NMF analysis was conducted for the data of protein-coding genes. Other 127 LUSC microarray samples (Affymetrix Human Genome U133 Plus 2.0 Array) of patients from two independent datasets were utilized for external validation. The survival data in two independent datasets, including GSE30219 [15] and GSE37745 [16], were acquired through GEOquery [17] in the R package (https://www.r-project.org). The data for clinical outcomes and gene expression profiles of 28 melanoma patient samples (GSE78220) with anti-PD-1 therapy was retrieved from GEO [18]. These samples included 15 responding and 13 non-responding pre-treatment tumours and were profiled by RNAseq. Their response patterns were based on irRECIST [19]. More details for these datasets are listed in Additional file 2: Table S1. The software tools used in this study are summarized in Additional file 2: Table S2.

### Identification of exhausted immune class by unsupervised clustering

**Fig. 1:**
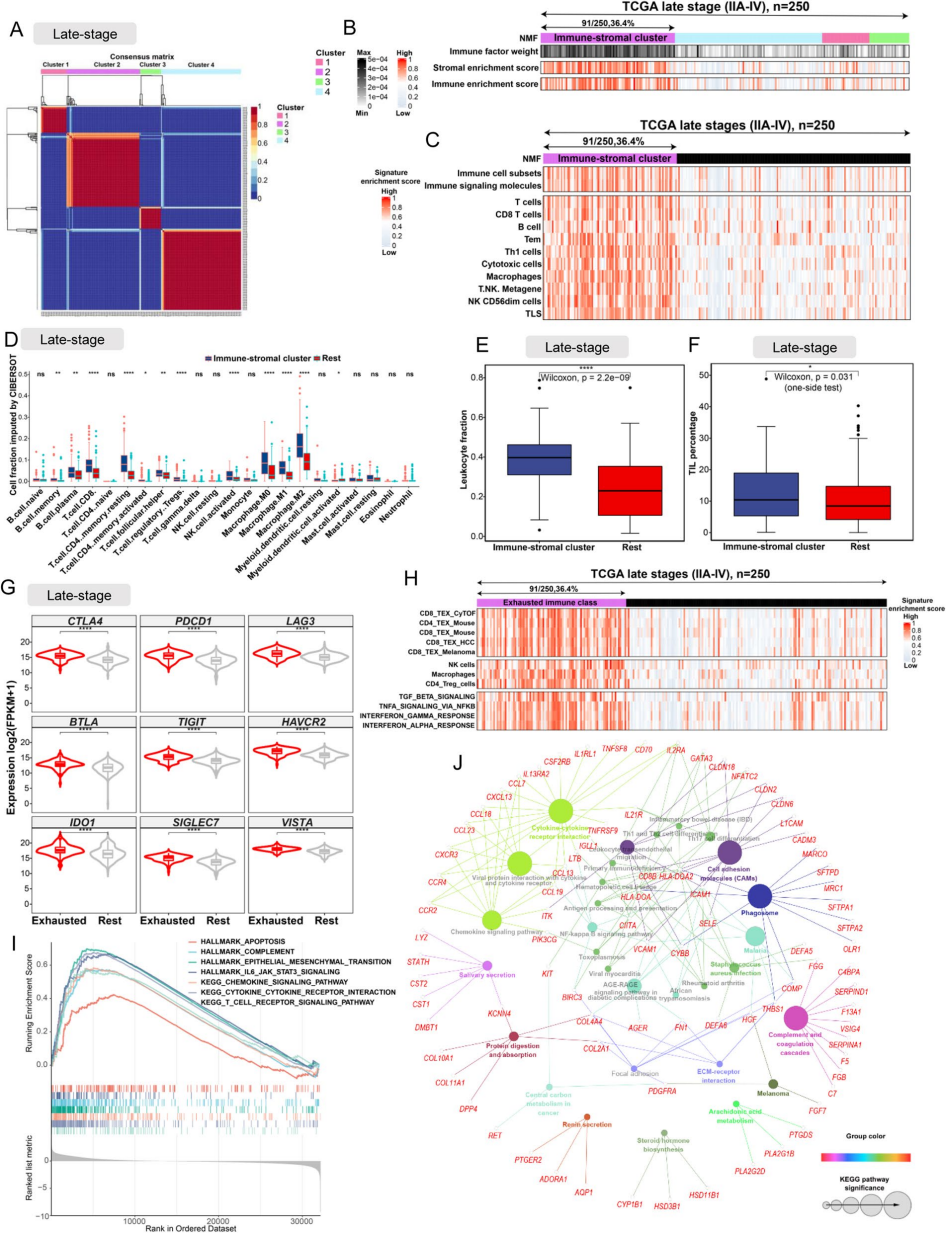
The identification and molecular characterization of EIC. **A** The heatmap of gene expression clusters for 250 late-stage (IIA-IV) LUSC samples by unsupervised NMF illustrates 4 distinct expression patterns. **B** Stromal and immune enrichment analysis defined the cluster 2 of four expression patterns as an immune-stromal cluster. High and low gene enrichment scores are delineated in red and grey, respectively. **C** The enrichment scores of gene signatures identified the immune cells for the immune-stromal and other clusters. **D** The comparison of the absolute fractions of TME cells inferred by CIBERSORT between two classes. **E,F** Box plots show the differences of leukocyte fraction and TIL percentage between two classes. **G** Box plots show different expression levels of multiple inhibitory receptors in the immune-stromal cluster compared to the other clusters. **H** The consensus-clustered heatmap of 250 LUSC samples defined the immune-stromal cluster as exhausted immune class (EIC). High and low gene enrichment scores are represented in red and grey, respectively. **I** GSEA analysis indicated the EIC showed significant enrichments of hallmark gene sets and KEGG pathways related to cytokine, T cell receptor, epithelial mesenchymal transition, and apoptosis. **J** The functionally grouped network of KEGG pathways by ClueGO/CluePedia for the interpretation of metagene-specific genes’ biological roles. Colourless and colour nodes represent metagene-specific genes and KEGG pathway terms, respectively. Node colours represent distinct functional groups. Node size represents the significance of KEGG pathways. The more significant KEGG pathways are, the larger highlighted nodes. All statistical differences of two classes were compared by Wilcoxon rank-sum test; *, *P* < 0.05; **, *P* < 0.01; ***, *P* < 0.001; ****, *P* < 0.0001

A virtual microdissection analysis was firstly conducted on the bulk RNAseq-based gene expression profiles of a training cohort of 250 patients using NMF in R [13, 20] (Additional file 1: Fig. S1). The factorization rank *r* which defines the number of clusters is the most critical parameter in NMF. When *r* equalled 4, the highest cophenetic correlation coefficient (Additional file 1: Fig. S2A) was obtained and the TCGA training cohort dataset was effectively decomposed (Fig. 1A) [20]. Therefore, *r* was set to be 4 in this study. Following the approach in a previous study [12], the immune and the stromal enrichment scores were calculated by the single sample gene set enrichment analysis (ssGSEA) [21, 22], which was wrapped in GSVA [23] to uncover immune and stromal-related expression patterns. When integrating the immune and stromal enrichment scores with the 4 NMF-identified clusters, we observed that Cluster 2 had higher enrichment scores (Fig. 1B) compared with other clusters. Therefore, Cluster 2 herein was referred as the ‘immune-stromal cluster’.

Secondly, we wanted to investigate the abundance of specific immune cells in tumours belonging to the immune-stromal cluster. A number of signatures representing various immune cells (Additional file 2: Table S3) were collected and used to compute the enrichment scores based on the expression profiles by ssGSEA. Then the enrichment scores of immune cells were integrated with the clusters to identify the abundance of specific immune cells in the immune-stromal cluster. In addition, the absolute fraction data of 22 infiltrating immune cells, which was inferred by the CIBERSORT algorithm [24] based on gene expression profiles, was downloaded from the TIMER database [25] (http://timer.cistrome.org/infiltration_estimation_for_tcga.csv.gz). And the leukocyte fraction data (TCGA_all_leuk_estimate.masked.20170107.tsv), which was estimated based on DNA methylation in Thorsson’s study [26], was retrieved from https://gdc.cancer.gov/about-data/publications/panimmune. The tumour-infiltrating lymphocyte (TIL) percentage, which was evaluated through pathological images of TCGA tumours including LUSC, can be found in the supplementary table (Table S1) in Saltz’s study [27]. The absolute fraction data of 22 infiltrating immune cells, the leukocyte fraction, and the TIL percentages for TCGA LUSC patients were extracted and then compared between the immune-stromal cluster and the rest clusters to verify the enrichment of lymphocytes in the immune-stromal cluster by Wilcoxon rank-sum test.

Finally, the expression profile analysis of multiple inhibitory receptors was performed, and the gene signatures representing T cell exhaustion were scored by ssGSEA. We observed that the immune-stromal cluster overexpressed multiple inhibitory receptors and had high T cell exhaustion-related signature enrichment scores. Consequently, we defined the patient population within the immune-stromal cluster as the exhausted immune class (EIC) and the rest population as the rest class. We used the method extractFeature [28] wrapped in NMF to extract the relevant genes (named as metagene-specific genes) in order to characterize the EIC expression pattern.

### Molecular characterization of exhausted immune class

The analyses by GSEA [22] and ssGSEA were conducted to evaluate the enrichment of molecular pathways and gene expression signatures in the EIC. The data of gene expression signatures was collected from previous studies [29–32] to represent distinct immune cells and exhausted T cells involved in various diseases (e.g. hepatocellular carcinoma, melanoma, and chronic infection). The hallmark gene sets and the KEGG pathway signatures were collated from MSigDB (https://www.gsea-msigdb.org/gsea/msigdb). ClueGO [33], a Cytoscape [34] plugin, was applied to generate the functionally grouped network of KEGG pathways to interpret the biological roles of metagene-specific genes. To identify the differentially expressed genes between the EIC and the rest class, the reads-count data of the training cohort was downloaded from TCGA. DESeq2 was used for gene differential expression analysis with false discovery rate (FDR) less than 0.05 and log2 fold change (log2FC) greater than 1 [35]. Of the metagene-specific genes, the differentially expressed genes (FDR < 0.05, log2FC ≥ 1) between the EIC and the rest class were defined as the exhausted immune classifier genes for identifying the EIC in the training cohort from TCGA. GSEA was applied to identify activated pathways and hallmark gene sets enriched in the EIC.

### Internal validation of EIC in early-stage TCGA LUSC

To confirm the presence of exhausted immune status in early-stage LUSC, we performed NMF and ssGSEA analysis on the bulk RNAseq-based expression profiles of 247 early-stage samples by using the same approach described above. Similarly, we also obtained 4 clusters for the early-stage LUSC cohort. When integrating the enrichment scores of early-stage samples for the signatures used in the late-stage LUSC cohort, we also observed that Cluster 2 had higher immune cell, stromal, and TEX-related signature enrichment scores. Therefore, Cluster2 was identified as EIC for the early-stage LUSC. The proportion of immune cells, leukocyte fraction, TIL percentage, and the expression of multiple inhibitory receptors were compared between the EIC and the rest class in these samples. GSEA was conducted to identify the enrichment of hallmarks and KEGG pathways. We used ssGSEA to calculate the enrichment score of 167 exhausted immune classifier genes obtained in the training stage and defined it as the EIC score. Receiver operating characteristic (ROC) analysis was utilized to evaluate the predictive capacity of the EIC score.

### Prediction of ICB therapy for EIC

The tumour immune dysfunction and exclusion (TIDE) algorithm [36] was used to predict potential ICB therapy response. We also retrieved the public data of melanoma tumour expressions and the clinical outcomes of patients treated with anti-PD-1 [37] to validate the association between ICB therapy resistance and 167 exhausted immune classifier genes. Specifically, the EIC scores of melanoma tumour samples were calculated by ssGSEA using the 167 exhausted immune classifier genes and compared between the responders and the non-responders.

### Analysis of genomic mutation, chromosomal alteration, and methylation profile for EIC

Maftools [38] was used to visualize and analyse somatic mutations and the total number of somatic mutations was counted. The statistical information of neoantigens for LUSC patients was obtained from Rooney’s study [39]. The copy number data generated by GISTIC2.0 [40] for TCGA LUSC samples was retrieved from the cBioPortal for Cancer Genomics database (http://www.cbioportal.org), followed by the data extraction for the copy number alterations of cytobands and focal genes. Specifically, the amplification and deletion data for cytobands of each LUSC sample was extracted from the file ‘all_lesions.conf_99.txt’, and the amplification and deletion data for focal genes was extracted from the file ‘all_thresholded.by_genes.txt’. Both files were generated by GISTIC2.0 and available from http://www.cbioportal.org. Then we assessed the difference in somatic mutations, the number of neoantigens, and the copy number alterations between the EIC and the rest class.

The genomic methylation data for 367 LUSC patient samples of all tumour stages was obtained from TCGA. The 367 patients were a subset of 497 patients used in RNAseq data analysis. The methylation CpGs’ Beta-value data of different samples was merged into a Beta-value matrix in which columns corresponded to samples and rows to CpGs. To correct the probe design bias in the Illumina Infinium 450K DNA methylation data, the Beta-value matrix was normalized by the Beta-mixture quantile normalization method using ChAMP in R [41]. We then performed a linear model using ‘limma’ in R to identify the significant CpG sites (FDR adjusted *p*-value < 0.05) that were differentially methylated between the EIC and the rest class identified by transcriptome analysis (deltaBeta >0.2). We only selected differentially methylated CpG sites located in the promoter regions of differentially expressed genes in the EIC compared with the rest class. The selected CpG sites were used to generate and cluster supervised heatmaps based on the Euclidean distances aggregated by Complete-linkage. Finally, the correlations between the methylation level of promoter regions and the corresponding gene expression were computed.

### Validation of EIC in independent datasets

The robustness of exhausted immune classifier genes was evaluated by an unsupervised Random Forest procedure [42]. Based on the expression value of these genes, we used the randomForest R package to perform an unsupervised learning on the training cohort with the parameter ‘ntree = 500’, and then visualized the samples distribution through the function ‘MDSplot’ wrapped in this package. The ability of exhausted immune classifier genes to predict exhausted immune status was validated in two additional datasets by using NMF. The gene signatures related to T cell exhaustion (Additional file 2: Table S3) were applied to validate and characterize the EIC captured by the classifier in each dataset.

### Highly visual interactive web application

Based on the analysis data in this study, we used ‘shiny’ in R to build an interactive web application (http://lilab2.sysu.edu.cn/tex & http://liwzlab.cn/tex) for other researchers to explore potential mechanisms of immunotherapy resistance at multi-omics level. The web application has included several immunotherapy resistance-related analysis modules, such as exhausted immune classifier module, signature expression module, somatic mutation module, clinical prognosis module, microRNA module, and methylation module. The source codes generated to build the web application are available at the GitHub repository (https://github.com/Lilab-SYSU/LUSC_Tex) [43].

### Protein expression analysis

To investigate protein expression alterations in EIC, the protein expression data of 319 LUSC patients was downloaded from TCGA. This data included 487 proteins profiled by reverse-phase protein arrays (RPPAs). The 319 LUSC patients were a subset of 497 patients for the training and internal validating cohort used in the bulk RNAseq data analysis. Then the protein expression was compared between the EIC and the rest class. The significantly upregulated proteins in EIC were identified. Moreover, we explored these protein expression profiles of LUSC on the immunohistochemically stained images via the pathology section of the Human Protein Atlas (HPA; https://www.proteinatlas.org/) [44]. Specifically, we searched the genes in HPA and found 12 LUSC patient samples which had the quantity information of the corresponding protein expressions in immunohistochemically stained images. Typical images were then selected to demonstrate the IDO protein expression level.

### Statistical analysis

All statistical discrete analyses were performed in SPSS (version 19.0, IBM) and R (version 3.5.1, http://www.r-project.org). We correlated the EIC and the rest class with TIL percentage, copy number alteration, mutation number, and neoantigen number by the Wilcoxon rank-sum test for continuous data. Kaplan–Meier estimate and log-rank testing were used to perform the survival analysis for overall survival (OS) and progression-free survival (PFS). We incorporated all clinicopathological variables into Cox model to identify the combination of variables. Two-tailed or one-tailed *P*-values < 0.05 were considered statistically significant. Pearson correlation was applied to measure the strength of the linear relationship between two variables.

## Results

### Identification and characterization of a novel exhausted immune class in late-stage LUSC

We performed a NMF analysis on the bulk RNAseq-based gene expression profiles of 250 late-stage LUSC samples in the training cohort and isolated the transcriptomic signals related to TEX in TME. The dataset of training cohort was effectively separated into four expression clusters (Fig. 1A). LUSC patients in cluster 2 possessed both high immune and stromal enrichment scores calculated by ssGSEA with bulk RNAseq-based gene expression profiles [12], indicating the significant enrichment of immune cell and stromal component gene expression signatures. Subsequently, we herein named this group as the immune-stromal cluster (Fig. 1B). The immune-stromal cluster also showed the significant enrichment of the signatures of immune cells (Fig. 1C), including immune cell subsets, T cells, B cells, macrophages, tertiary lymphoid structures (TLS), Tem, th1 cell, cytotoxic cells, and T.NK. metagene (all, *P* < 0.001). To further verify the enrichment of these immune cells in the immune-stromal cluster, the absolute proportions of immune cells, which were imputed by CIBERSORT using bulk RNAseq data, were compared between the immune-stromal and the rest clusters. Consistent with our enrichment analysis by ssGSEA, the immune-stromal cluster had higher proportions of CD8 T cells, macrophages, and B cells than the rest clusters (Fig. 1D). Moreover, the leukocyte fraction estimated by DNA methylation [26] was significantly higher in the immune-stromal cluster (median fraction 0.40) than that in the rest clusters (median fraction 0.23) (Fig. 1E). Similarly, the tumour-infiltrating lymphocytes (TILs) evaluated through the pathological images [27] were significantly higher in the immune-stromal cluster (median TILs percentage 10.35) than that in the rest clusters (median TILs percentage 8.25) (Fig. 1F).

To explore TEX in LUSC, we carried out an expression profile analysis of multiple inhibitory receptors, such as *CTLA4*, *PDCD1* (known as *PD-1*), *LAG3*, *BTLA*, *TIGIT*, *HAVCR2* (known as TIM-3), *IDO1*, *SIGLEC7*, and *VISTA*. These inhibitory receptors were significantly upregulated in the tumour samples within the immune-stromal cluster (fold-change > 2, Benjamini-Hochberg false discovery rate [FDR] < 1.1 × 10^−7^) (Fig. 1G). Meanwhile, patient samples within the immune-stromal cluster showed a significant enrichment of multiple gene sets for identifying TEX (Fig. 1H), such as CD8 TEX revealed by mass cytometry profiling (CyTOF) (CD8_TEX_CyTOF, *P* < 0.001) [29], human gene sets homologous to CD4 TEX (CD4_TEX_Mouse, *P* < 0.001) and CD8 TEX (CD8_TEX_Mouse, *P* < .001) in mice with chronic viral infection [30], CD8 TEX in hepatocellular carcinoma (HCC) (CD8_TEX_HCC, *P* < .001) [45], and CD8 TEX in melanoma patients (CD8_TEX_Melanoma, *P* < 0.001) [32].

Based on the above expression analysis of inhibitory receptors and the enrichment scores of TEX signal gene sets, we identified a new population subgroup belonging to the immune-stromal cluster, accounting for 36.4% of the training cohort (91/250), referred herein as the exhausted immune class (EIC) (Fig. 1H). The rest subpopulation group in the training cohort was defined as the rest class. To further validate the T cell exhaustion of EIC, we collected 3 gene signatures which characterize three immunosuppressive cell types (NK cells, macrophages, and CD4_Treg_cells) involved in TEX regulation, and 4 hallmark gene sets of cytokines that promote TEX [31] (Fig. 1H). The high enrichment scores of the above gene sets verified the immunosuppressive TME of the EIC. The above results demonstrated that we successfully identified an exhausted immune class, which showed a significant enrichment of gene expression signatures of exhausted T cells and other immunosuppressive cells in TME.

### Cytokine enrichment is a specific molecular feature of EIC

To characterize molecular features of EIC, gene set enrichment analysis (GESA) based on the gene expression profile of the training cohort identified 48 KEGG pathways (Additional file 1: Fig. S2B) and 17 hallmark gene sets (Additional file 1: Fig. S2C) with enrichment in the EIC. Particularly, cytokine-related pathways and hallmarks, such as cytokine and cytokine receptor interaction pathway, chemokine signalling pathway, and complement hallmark (Fig. 1 I), were significantly enriched in the EIC. Previous studies suggested that severely exhausted T cells may suffer from apoptosis [46, 47], and our analysis showed that apoptosis hallmark was significantly enriched in the EIC. These implied severe T cell exhaustion in the EIC of late-stage LUSC.

In total, 253 metagene-specific genes representing the EIC expression pattern were extracted by NMF and grouped into a network of KEGG pathways by ClueGO (Fig. 1 J) to further reveal their molecular functions. Most of these genes were also associated with cytokine and its complements, which mediate immune response and inflammatory response. Transcriptomics comparison between the EIC and the rest class identified 167 significantly different genes (FDR < 0.01 and fold-change > 2), which were a part of the metagene-specific genes aforementioned. These genes were defined as exhausted immune classifier genes that can be used to confirm the exhausted immune status in LUSC, including 165 upregulated genes related to immunosuppression, such as chemokine and chemokine receptors (*CCL13*, *CCL18*, *CCR4*, *CCR2*, *CXCR3*, *CCL7*, and *CCL19*), interleukin receptor molecules (*IL2RA*, *IL21R*, and *IL17REL*), tumour necrosis factor-related molecules (*TlNFSF8* and *TNFRSF9*), complement-related molecules (*C7* and *C4BPA*), and *WNT6* (FDR < 0.001) (Additional file 2: Table S4). The above characterization of molecular functions indicated that cytokine-related expression signals played a dominant role in the exhausted immune status of LUSC.

### Internal validation of EIC in early-stage TCGA LUSC

**Fig. 2:**
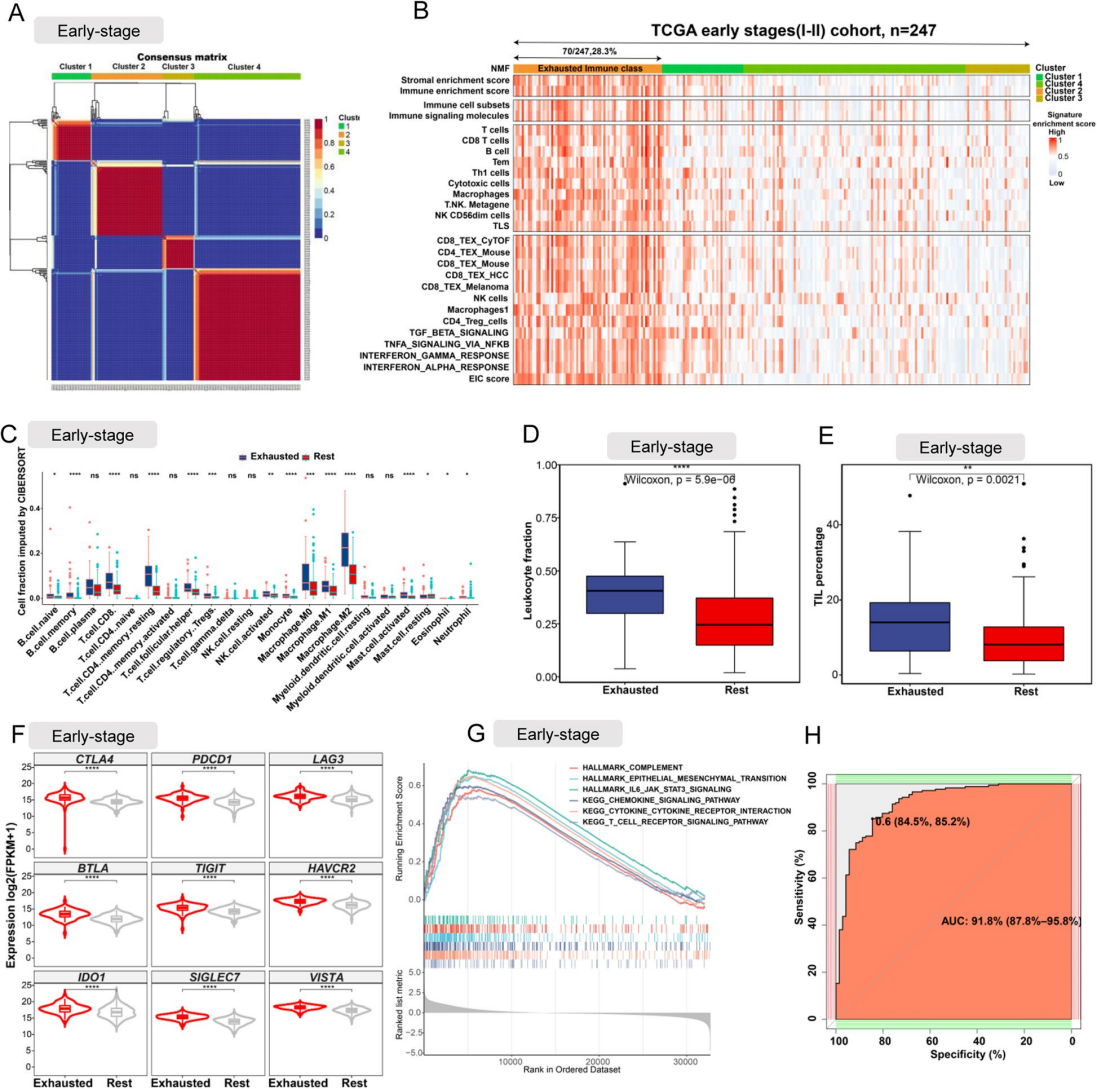
Internal validation of EIC on 247 early-stage (I–II) LUSC samples. **A** The heatmap of gene expression clusters for 247 early-stage (IIA–IV) LUSC samples by unsupervised NMF illustrates 4 distinct expression patterns. **B** Heatmap shows the cluster 2 (defined as EIC) exhibited high enrichment scores of gene signatures of T cell exhaustion, immunosuppressive cells, and immunosuppressive cytokine. **C** The comparison of the absolute fraction of TME cells between the EIC and the rest class. **D,E** Box plots show the differences of leukocyte fraction and TIL percentage between two classes. **F** Box plots shows the different expression levels of multiple inhibitory receptors between two classes. **G** Cytokine-, T cell receptor-, and epithelial mesenchymal transition-related hallmark gene sets and KEGG pathways enriched in the EIC. **H** ROC curve evaluated the predictive capacity of 167 exhausted immune classifier genes in early-stage LUSC samples. All statistical differences of two groups were computed by Wilcoxon rank-sum test; *, *P* < 0.05; **, *P* < 0.01; ***, *P* < 0.001; ****, *P* < 0.0001

To verify whether exhausted immune class existed in early-stage LUSC, we performed NMF on the bulk RNAseq-based gene expression profiles of the additional 247 early-stage TCGA LUSC samples and subsequently obtained 4 clusters (Fig. 2A). When integrating the clusters with signature enrichment scores calculated by ssGSEA with bulk RNAseq-based gene expression profiles, we observed that stromal and immune enrichment scores of Cluster 2 were higher than those of other clusters (Fig. 2B). Moreover, Cluster 2 also showed a significant enrichment of gene expression signatures of immune cells (CD8 T cells, T cells, macrophages, Tem, th1 cell, cytotoxic cells, NK cell, and exhausted CD8 T cells [all, *P* <0.001]) and other cytokine hallmarks related to TEX (Fig. 2B). Therefore, Cluster 2 was defined as the Exhausted Immune class in the early-stage TCGA LUSC samples. Consistent with the EIC in the late-stage LUSC, the samples in the EIC of early-stage LUSC had higher proportions of immune cells (CD8 T cells, macrophage, and NK cells) imputed by CIBERSORT (Fig. 2C), higher leukocyte fraction (Fig. 2D), and higher tumour-infiltrating lymphocytes percentage (Fig. 2E) than the rest samples. And co-upregulation of multiple inhibitory receptors was also observed in the EIC of early-stage LUSC (Fig. 2F). GSEA analysis showed significant enrichments of cytokine pathways and complement hallmarks in the EIC of early-stage LUSC. However, we did not observe enrichment of apoptosis hallmark in the EIC of early-stage LUSC, suggesting that the EIC of early-stage LUSC had a lower level of TEX than the EIC of late-stage LUSC. The ROC curve with an AUC of 0.918 (Fig. 2H) showed that our 167 exhausted immune classifier genes had good performance in predicting EIC.

### EIC had poorer prognosis in late-stage LUSC

**Fig. 3:**
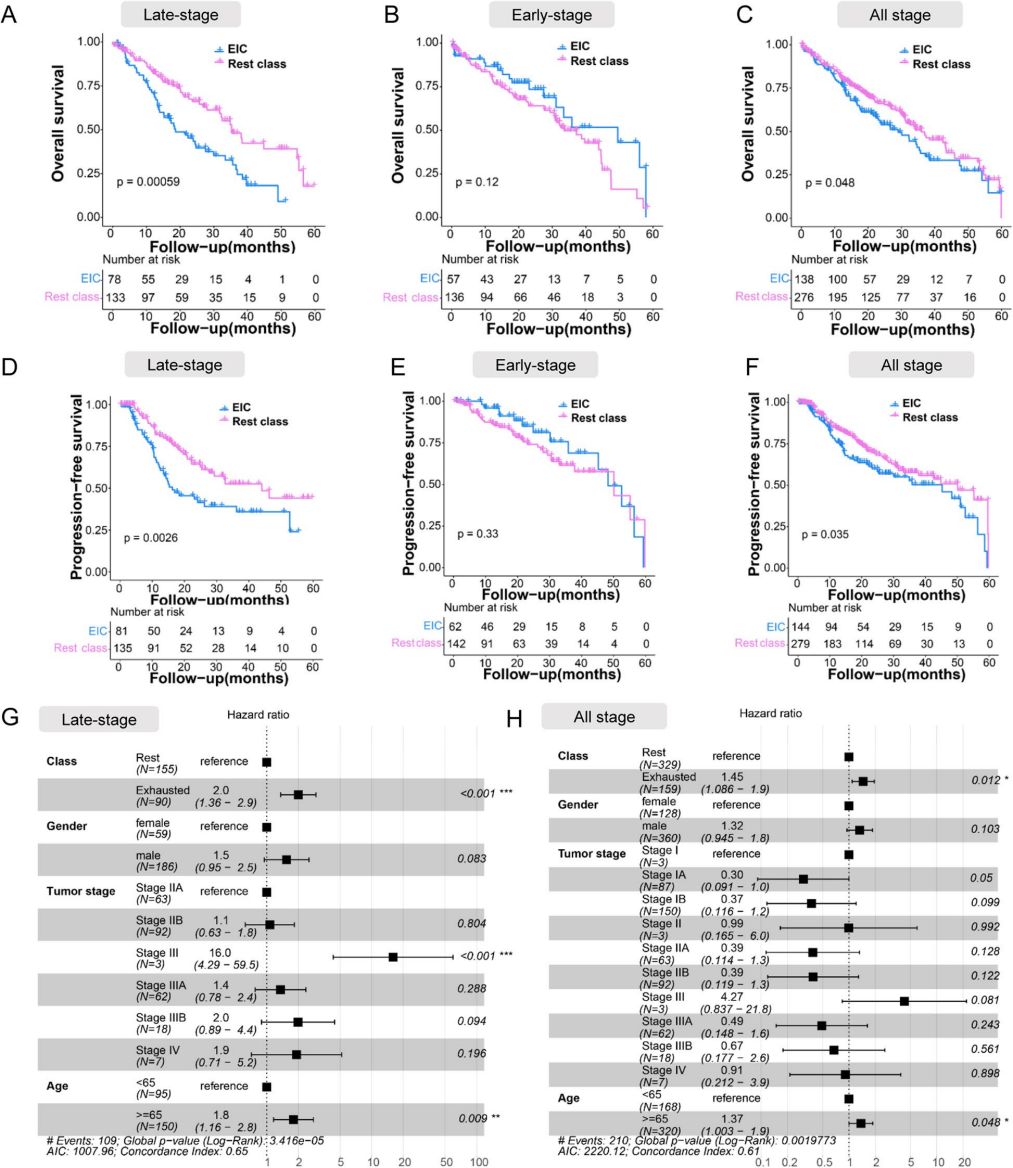
Prognosis analysis for the EIC and the rest class across different stages of LUSC. **A–C** Kaplan–Meier estimates of overall survival for the EIC and the rest class across late-stage, early-stage, and all-stage LUSC. Survival data was limited to maximum 5 years (60 months). **D–F** Kaplan–Meier estimates of progression-free survival for the EIC and the rest class across late-stage, early-stage, and all-stage LUSC. *P*-values were calculated by log-rank test. Survival data was limited to maximum 5 years (60 months). **G,H** Multivariate Cox regression analysis on four variables (class, gender, tumour stage, and age) for late-stage and all-stage LUSC

We investigated the prognostic value of exhausted immune status by correlating the classes with clinicopathologic variables. Previous studies suggested that the high densities of TILs correlated with favourable prognoses such as longer progression-free survival (PFS) or improved overall survival (OS) [48, 49]. In our study, the higher percentage of TILs was observed in the EIC compared with the rest class in both late-stage and early-stage LUSC (Figs. 1 F and 2E). However, in the late-stage LUSC, Kaplan–Meier estimates demonstrated that patients within the EIC had significantly poorer OS (*P* < 0.001; Fig. 3A) and PFS (*P* < 0.01; Fig. 3D) than the rest class. Multivariate survival analysis using the Cox regression model indicated that the EIC was retained as an independent prognostic factor for OS (*P* <0.001) in the late-stage LUSC (Fig. 3G). With regard to the early-stage LUSC, there was no difference of both OS and PFS between the EIC and the rest class (Fig. 3 B,E). Finally, we investigated prognostic value in all stage LUSC patients. As expected, patients within the EIC exhibited worse OS and PFS than within the rest class (Fig. 3 C,F). These results verified that although abundant T cells existed in the EIC, the majority of T cells were in the immunosuppressive status and lost the effector function to control tumour progress, resulting in poorer prognosis. The survival results also verified that the EIC in the late-stage LUSC showed more severe T cell exhaustion than in the early-stage LUSC.

### EIC is associated with immunotherapy resistance

**Fig. 4:**
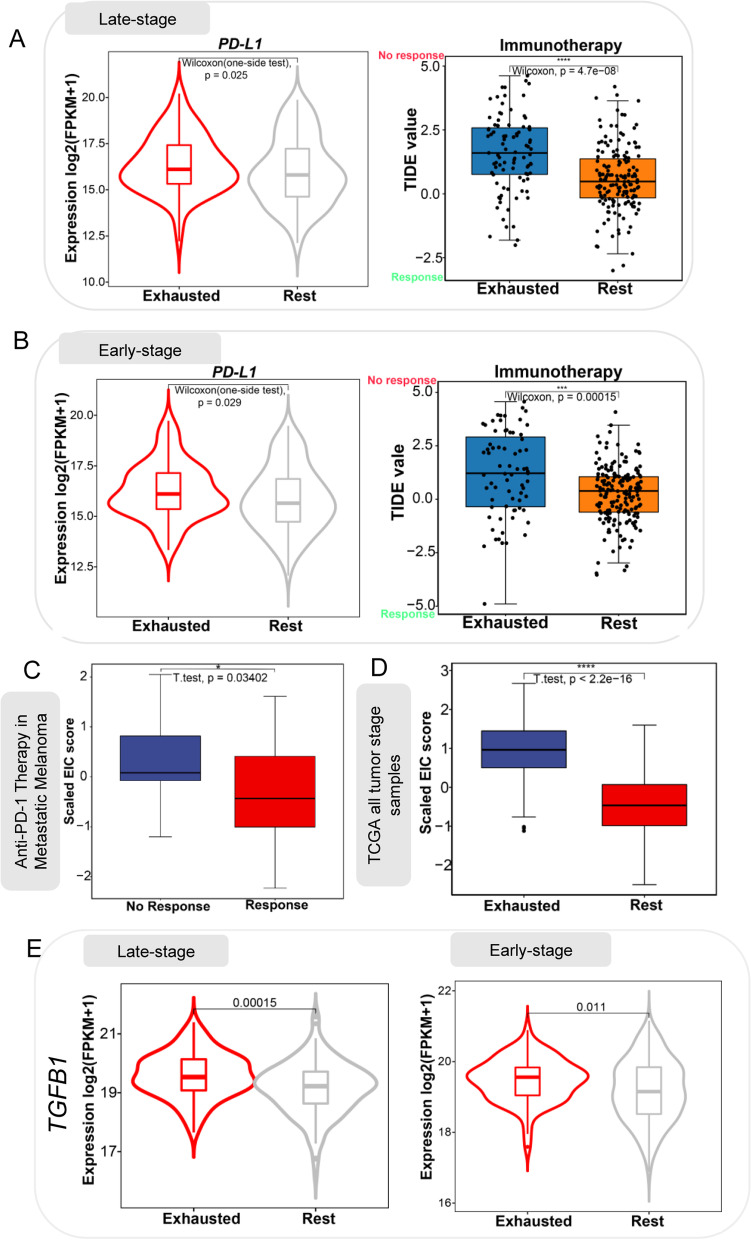
Prediction of ICB therapy resistance. **A,B** Patients in the EIC showed a relatively higher expression level of PD-L1 and higher TIDE prediction score for ICB therapy. **C** Metastatic melanoma patients with no response to anti-PD-1 therapy had higher enrichment scores of 167 exhausted immune classifier genes compared to patients with response. **D** The EIC showed higher enrichment score than the rest class in all tumour stage TCGA LUSC. **E** Box plot shows higher expression of TGFB1 in the EIC than rest class

To investigate the response of patients within the EIC to ICB therapy, we compared PD-L1 expression between the EIC and the rest class and found that the EIC had a higher expression level of PD-L1 than the rest class in both early-stage and late-stage LUSC (Fig. 4 A,B). We also used the tumour immune dysfunction and exclusion (TIDE) algorithm [36] to predict ICB therapy response and observed that the EIC had higher TIDE prediction scores than the rest class in both early-stage and late-stage LUSC (Fig. 4 A,B). A higher TIDE prediction score is usually associated with worse ICB response. Our result suggested that although patients within the EIC had high *PD-L1* expression, they were possibly resistant to ICB therapy.

To further validate the immunotherapy resistance of EIC, we calculated the enrichment score of 167 exhausted immune classifier genes on the RNAseq data of 28 metastatic melanoma patients (GEO: GSE78220) treated with anti-PD-1 [18] and 497 LUSC patients of all tumour stages from TCGA (Project ID: TCGA LUSC, dbGaP Study Accession: phs000178) [14]. We found the melanoma patients who did not respond to ICB therapy had higher enrichment scores than those who respond to ICB therapy (Fig. 4C). As expected, the patients within the EIC also showed higher enrichment scores than the rest class, further evidencing the resistance of EIC (Fig. 4D). We observed a higher expression level of TGFB1 in the EIC than in the rest class (Fig. 4E), and this is consistent with Mariathasan’s study which found cytokine TGFβ (encoded by TGFB1) suppressed anti-tumour immunotherapy [50]. These all suggested the ICB therapy resistance of EIC.

### EIC has distinctive methylation patterns

**Fig. 5:**
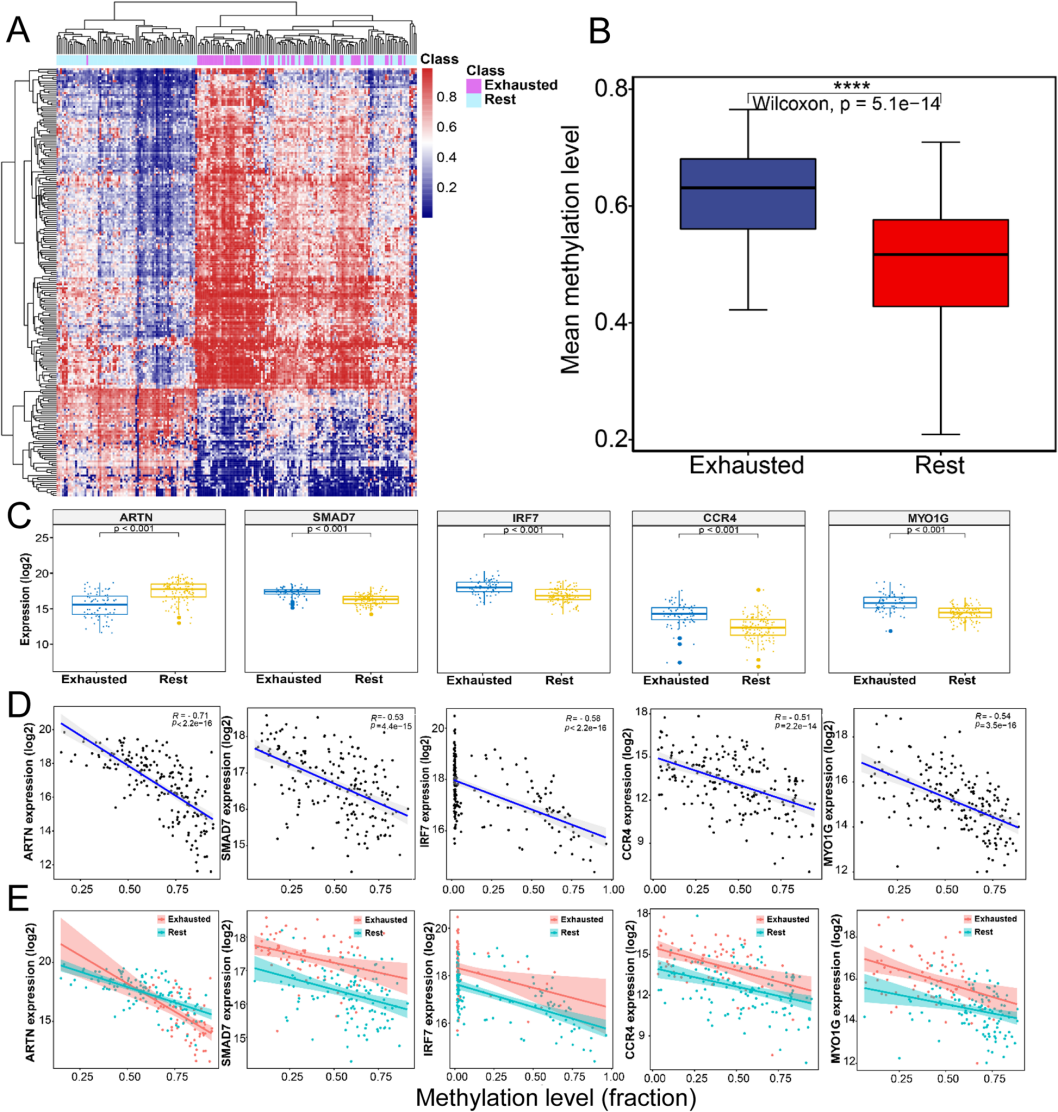
Distinctive methylation signatures characterized the EIC of LUSC. **A** Hierarchical clustering heatmap of 216 CpG methylation values located within 162 immunosuppression-related gene promoters show significant difference between the EIC and the rest class (FDR< 0.05, diff > 0.2). **B** Boxplot displays the mean methylation levels of 216 CpG sites within 162 immune exhaustion-related gene promoters for 2 classes. Wilcoxon rank-sum test (*p*<0.0001). Exhausted vs Rest: *p*=5.1E−15. **C–E** Correlations between expression and promoter methylation levels for deregulated genes in the EIC to the rest class. **C***ARTN* exhibited significantly lower expression in the EIC, while *SMAD7*, *IRF7*, *CCR4*, and *MYO1G* were synergistically overexpressed (*P* < 0.001, Wilcoxon rank-sum test). **D** The expressions of *ARTN*, *SMAD7*, *IRF7*, *CCR4*, and *MYO1G* negatively correlated with their promoter methylation level for the whole training cohorts. **E***SMAD7*, *IRF7*, *CCR4*, and *MYO1G* had lower promoter methylation levels mirroring higher expression levels in the EIC, whereas *ARTN* had an opposite status. Red dots in the plotting represent the members of EIC, and blue dots represent the members of the rest class

De novo DNA methylation programs can promote T cell exhaustion, and blocking these programs can enhance exhausted T cell rejuvenation, aiding tumour control by immune checkpoint blockade [51]. To explore epigenetic alteration related to deregulated genes in the EIC, a whole-genome methylation profiling analysis on the TCGA cohort of all tumour stages was conducted and found that 216 CpG sites located in 162 immune-related gene promoter regions were differentially methylated in the EIC compared to the rest class (FDR<0.05) (Fig. 5 A,B and Additional file 2: Table S5). A total of 111 of 162 genes had significant correlations between methylation and gene expression (Additional file 2: Table S6). This indicated that the EIC showed specific methylation profiles, and most of the 162 genes were significantly associated with their promoter methylations.

In particular, multiple genes were regulated by their promoter methylations and involved in the TGF-β signalling pathway, which plays an important role in immune evasion [52] and immunotherapy resistance [50]. For example, *ARTN* is a ligand of transforming growth factor-beta (TGF-beta) superfamily proteins and binds various TGF-beta receptors, leading to the recruitment and activation of SMAD family transcription factors [53, 54]. We observed that *ARTN* exhibited a higher methylation level in its promoter region and a lower gene expression level in the EIC compared to the rest class (Fig. 5 C–E). On the contrary, the transcription factor *SMAD7*, which can be activated by TGF-β to attenuate or restrain immune cell activation [55], showed a lower methylation level and a higher expression in the EIC (Fig. 5 C–E). In addition, plasma membrane-associated class I myosin (*MYO1G*), C-C Motif Chemokine Receptor 4 (*CCR4*), and interferon regulatory factor 7 (*IRF7)* were also overexpressed and lowly methylated in the EIC (Fig. 5 C–E). *MYO1G* is abundant in T and B lymphocytes and mast cells [56], and *CCR4* is a novel-specific molecular target for immunotherapy in Hodgkin lymphoma, able to regulate the cell trafficking of various leukocyte types [57].

### EIC shows no difference in tumour mutational burden or number of neoantigens, but has a lower burden of copy number alterations

**Fig. 6:**
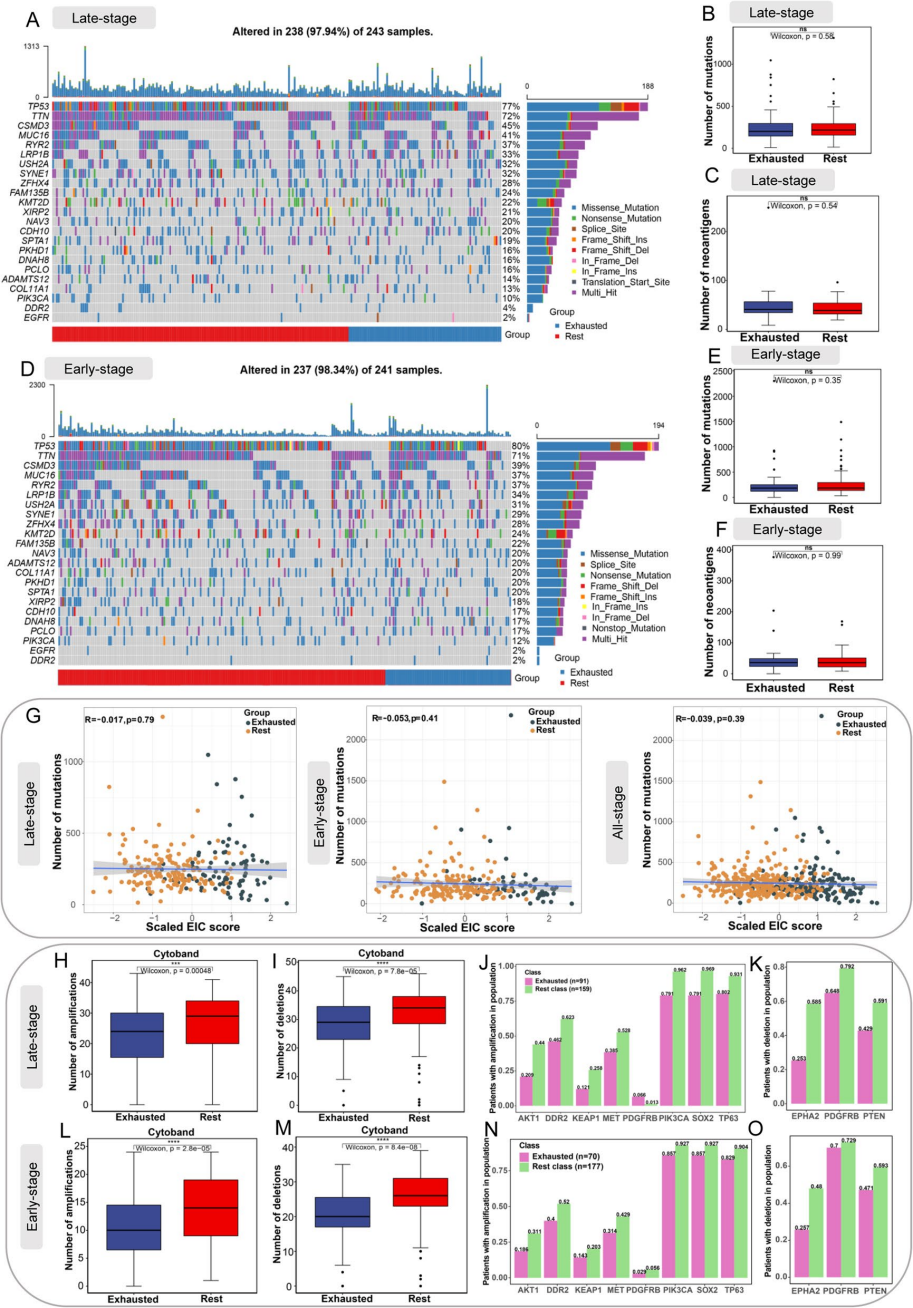
Association of EIC with somatic mutations, neoantigens, and copy number alteration. **A, D** The landscape of most frequently mutated genes between the EIC and the rest class in late-stage and early-stage LUSC, respectively. **B, E** Box plots show the number of mutations between two classes in late-stage and early-stage LUSC, respectively. **C, F** Box plots show the number of neoantigens between two classes in late-stage and early-stage LUSC, respectively. **G** Pearson correlation analysis between scaled the EIC score and the number of mutations across LUSC cohorts of late-stage, early-stage, and all-stage, respectively. **H, L** Box plots show significant difference of amplification burden of cytoband between two classes in late-stage and early-stage LUSC, respectively. **I, M** Box plots show significant difference of deletion burden of cytoband between two classes in late-stage and early-stage LUSC, respectively. For late-stage (**J**) and early-stage (**N**) LUSC, the frequency of patients with amplification of driver genes in two classes. For late-stage (**K**) and early-stage (**O**) LUSC, the frequency of patients with deletion of driver genes in two classes. All statistical significances of two classes were computed by Wilcoxon rank-sum test; *, *P* < 0.05; **, *P* < 0.01; ***, *P* < 0.001; ****, *P* < 0.0001

Genomic mutations of tumours had a strong association with immunotherapy outcome [58, 59]. We depicted a landscape of commonly mutated genes between the EIC and the rest class in both late-stage and early-stage LUSC. There was no significant difference of individual gene mutation between the EIC and the rest class (Fig 6 A,D). We also linked the burden of somatic mutations, and mutated neoantigens with the exhausted immune status in LUSC. We did not observe significant changes of the burden of tumour mutations or neoantigens between two classes in both late-stage (Fig. 6 B,C) and early-stage (Fig. 6 E,F) LUSC. These results indicated that somatic mutations and relevant neoantigens showed no significant association with immunosuppressive TME. Recent studies on molecular characteristics of cancer patients treated with immune checkpoint inhibitors demonstrated that tumour mutational burden (TMB) and gene expression profile-based biomarkers, such as IFNγ-6-related and T cell-inflamed gene expression profiles, had a low correlation and thus were independently predictive of response [60–63]. These studies also indicated that these biomarkers combined with TMB could improve the prediction of response. In our study, the EIC score was calculated by ssGSEA based on gene expression profiles, and the EIC patients showed no difference in TMB compared with the rest class patients. Therefore, we investigated the correlation between the EIC score and TMB. As Fig. 6G displays, there was no significant correlation between the EIC score and TMB across the LUSC cohorts of late-stage (*R*=−0.017, *P*=0.79), early-stage (*R*=−0.053, *P*=0.41), and all-stage (*R*=−0.039, *P*=0.39), indicating that the EIC score was independently predictive of immunotherapy resistance. Both our result and the literature evidences supported that the combination of the EIC score and TMB may improve the prediction of response to immunotherapy.

On the other hand, we observed the samples of EIC showed lower burden of copy number alterations in cytobands. Specifically, the EIC had a lower number of cytoband amplifications than the rest class in both late-stage (Fig. 6 H) and early-stage LUSC (Fig. 6 L), and EIC had a lower number of cytoband deletions too (Fig. 6 I,M). With regard to the amplification (Fig. 6 J,N) or deletion (Fig. 6 K,O) of driver genes, we found these genes also had lower alteration frequency in the EIC than the rest class.

### External validation of the novel exhausted immune class across two independent datasets

To validate the presence of exhausted immune status in the training cohort of 250 LUSC samples, we performed unsupervised random forest clustering by using 167 exhausted immune classifier genes. As displayed in the MDS plot (Additional file 1: Fig. S3A), most of the patients were successfully separated into two clusters, which were consistent with the classifications for the EIC and the rest class. The ability of exhausted immune classifier genes to predict immune exhaustion status was estimated in two additionally independent testing datasets (*n*=127 LUSC samples). Similar to the training cohort, about 30–35% of LUSC samples were successfully identified as EIC across the testing datasets. Based on the gene expression profiles, the molecular characterization of the testing datasets also verified that the EIC showed both high immune and stroma enrichment scores.

Taking GSE30219 (*n*=61 LUSC samples) as an example, 19 (31%) LUSC samples were predicted as the EIC, which had remarkable enrichment of immune exhaustion signatures (CD8_TEX_CyTOF, CD4_TEX_Mouse, CD8_TEX_Mouse, CD8_TEX_HCC and CD8_TEX_Melanoma; all, *P* < 0.001), TGF-β signalling pathway promoting TEX (*P* < 0.001), IFN signatures (INFTERFERON_GAMA_RESPONSE and INTERFERON_ALPHA_RESPONSE; both, *P* < 0.001), and hallmark genes regulated by NF-kB in response to TNF (TNFA_SIGNALING_VIA_NFKB, *P* <0.001) (Additional file 1: Fig. S3B). Furthermore, Kaplan–Meier survival analysis showed that the immune exhaustion status was also correlated with poor prognosis (*P*=0.013) (Additional file 1: Fig. S3D). In another example of GSE37745 (*n*=66 LUSC), 23 (34%) patients also showed the enrichment of T cell exhaustion signatures and exhausted molecular features (Additional file 1: Fig. S3C). Kaplan–Meier survival analysis on the 66 patients based on immune exhaustion status indicated that 23 of the 66 patients tended poor prognosis (Additional file 1: Fig. S3E).

The correlation between the clinical outcomes and the immune exhaustion status suggested that exhausted immune cells cannot control the progression of the tumour, leading to deteriorated survival. The prediction of ICB response by TIDE for both GSE30219 and GSE37745 also suggested that the EIC showed potential resistance to ICB therapy (Additional file 1: Fig. S3F&G).

### IDO protein expression is higher in EIC

**Fig. 7:**
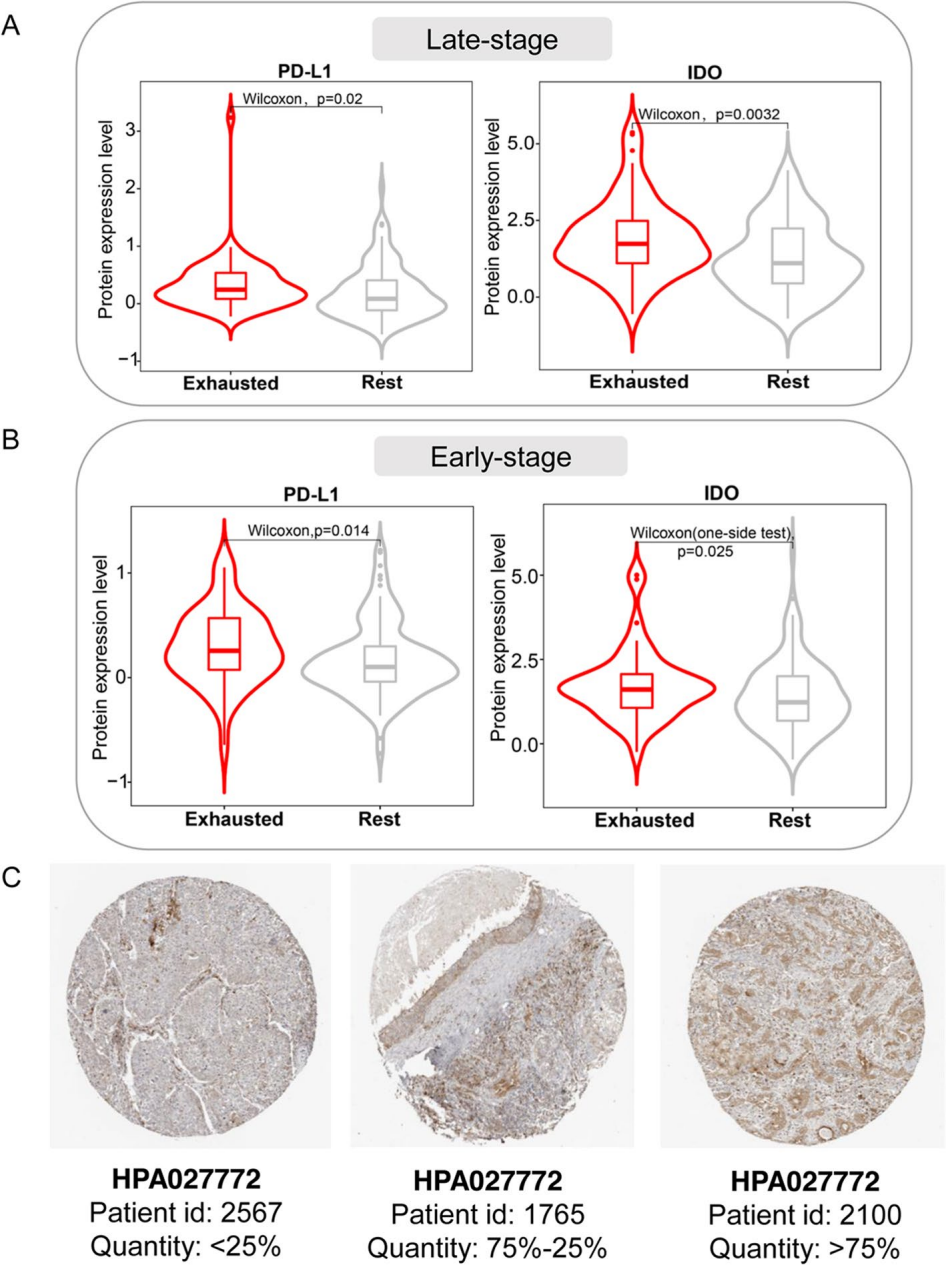
IDO protein expression analysis in EIC. **A, B** Boxplots showing protein expression difference between the EIC and the rest class in late-stage and early-stage LUSC, respectively. Wilcoxon rank-sum test was utilized to perform comparision analysis. **C** The immunohistochemically stained tissue images show different expression levels of IDO protein across three LUSC patients from HPA

We explored the difference in protein expressions between the EIC and the rest class using the RPPA data of LUSC. As expected, EIC showed significantly higher PD-L1 and IDO (Indoleamine-2,3-dioxygenase encoded by IDO1) protein expressions than the rest class in both late-stage (Fig. 7A) and early-stage (Fig. 7B) LUSC, consistent with the transcriptomic expression analysis aforementioned (Fig. 1G and Fig. 2 F). As well known, the evaluation of PD-L1 protein expression by immunohistochemistry was applied to select NSCLC patients to receive anti-PD-1 inhibitor treatment. Therefore, we investigated the IDO protein expression of different LUSC patients by immunohistochemically stained tissue images from HPA. We observed that LUSC patients showed different IDO immunohistochemical expression quantities. For example, Fig. 7 C shows three LUSC patients had different IDO expression quantities of less than 25%, between 25 and 75%, and more than75%, respectively. Additionally, high IDO activity was reported to be associated with primary resistance to immunotherapy in NSCLC [64]. These evidences indicated different IDO expressions of LUSC patients at the levels of transcriptomics, proteomics, and stained tissues, suggesting that IDO immunohistochemical expression may be a potential biomarker of immunotherapy resistance of LUSC with high PD-L1 expression.

## Discussion

Immunotherapy, especially ICB therapy, has revolutionarily transformed the treatment of LUSC and remarkably improved the overall survival of advanced-stage patients [5, 6]. However, more than half of the patients with high PD-L1 expression showed resistance to immune checkpoint inhibitors [6, 65]. Additionally, immune-related adverse events are frequent. Current understanding of the mechanisms of ICB therapy resistance remains limited. Immunosuppressive TME, which consists of tumour cells, immune cells, and other stromal components, may play a vital role in ICB resistance [65]. Thus, characterizing the molecular features of immunosuppressive TME is fundamental for identifying LUSC patients with ICB resistance and thus optimizing immunotherapy strategies.

In our study, we used NMF to deconvolute the gene expression signals derived from exhausted T cells, immune cells, and stromal components in TME of LUSC; then, we successfully identified a novel immunosuppressive class of LUSC (~30% of 624), herein defined as the EIC. Consistent with the exhausted immune class observed in head and neck squamous cell carcinoma [66] and hepatocellular carcinoma [67], our EIC had both high immune and stromal enrichment scores, which suggested the presence of abundant immune cells and stromal components. As expected, the EIC had specifically molecular features, including high immune cell infiltration, co-upregulation of multiple inhibitory receptors, enhanced immunosuppressive cytokine expression, and elevated PD-L1 expression. Of these immune cells, the M2 subtype of tumour-associated macrophages and CD4 Treg cells, as immunosuppressive cells, played a vital role in immune evasion and impacted ICB therapy [68, 69]. The EIC broadly existed in different tumour stages, but there was a potential difference at the T cell exhaustion level between early-stage (stages I–II) and late-stage (stages IIA to IV) LUSC. A previous study suggested that severely exhausted T cell may undergo apoptosis [70]. The apoptosis hallmark gene set was enriched in the EIC of the late stage, but not in the EIC of the early stage, suggesting that the EIC of the late stage had a higher T cell exhaustion level.

Previous observations indicated that high densities of TILs correlated with favourable prognosis [71]. Our EIC also had a high percentage of TILs. However, the EIC showed both poor overall survival and progression-free survival in late-stage LUSC; on the other hand, the EIC showed no significant difference in early-stage LUSC. This suggested that although there was a high T cell infiltration in TME, most T cells were in exhausted status and lacking of effector function to control tumour progress and to prolong survival time of the EIC patients. This also validated the EIC showed more severe immunosuppression in the late stage than in the early stage.

Clinical outcome analysis further confirmed that the EIC of LUSC had immunosuppressive TME. To understand the impact of immunosuppressive TME on ICB therapy, we used the TIDE algorithm to predict response to ICB therapy and observed that the EIC had higher TIDE prediction scores for resistance to ICB therapy [36]. Then 167 exhausted immune classifier genes for identifying EIC in LUSC were also significantly enriched in melanoma patients who showed no response to anti-PD-1 therapy. This further evidenced EIC’s potentiality of resistance to ICB therapy. A previous study indicated cytokine TGFβ promoted tumour immune evasion and resistance to ICB therapy [50], and the elevated expression level of *TGFB1* in our EIC also verified the potential resistance of the EIC patients.

Blocking de novo DNA methylation program may reactivate the effector function of exhausted T cell and improve the effectiveness of ICB therapy [51]. Our investigation of the epigenetic alterations indicated that the EIC exhibited a unique methylation pattern. Similar to a previous study in HCC [67], some exhaustion-related genes with differentially methylated promoters were involved in the TGF-β signal pathway. In particular, *ARTN* and *SMAD7* were differentially expressed in the EIC compared to the rest class, and their gene expressions were significantly correlated with their promoter methylation. *ARTN* encoded a secreted ligand of the TGF-β protein superfamily and its high expression was associated with the progression of NSCLC [72]. On the contrary, *ARTN* in our EIC had a lower expression level and a higher promoter methylation level, which were correlated with poor PFS. Additionally, a previous study suggested that the overexpression of *SMAD7* may suppress tumour progression by antagonizing TGF-β [73], while the high expression and low methylation of *SMAD7* in our EIC were associated with poor prognosis for LUSC. These suggested that there was a potential mechanism for regulating immunosuppressive TME by DNA methylation.

A previous study suggested that high tumour mutation burden and neoantigen load were related to the response to ICB therapy [74]. Interestingly, in our study, neither mutational burden nor neoantigen load showed a significant association with the EIC which has high lymphocyte infiltration. The EIC scores for predicting EIC were computed based on gene expression profiles; therefore, we reviewed recent studies on the association between TMB and gene expression profile-based biomarkers for predicting response to immunotherapy. Cristescu’s study on pan-tumour genomic biomarkers for predicting clinical response to PD-1 checkpoint blockade indicated that TMB and T cell-inflamed gene expression profiles were independently predictive of response and showed weak correlation [61]. KEYNOTE-028, a clinical trial to evaluate the associations between the biomarkers (e.g. TMB, PD-L1, and T cell-inflamed gene expression profile) and the clinical efficacy of pembrolizumab across 20 cancers, demonstrated that the correlations of TMB with gene expression profile and PD-L1 were low [62]. In addition, recent studies on the immunotherapy of melanoma exhibited that TMB and IFNγ-related gene expression show no significant correlation and both are able to predict response independently [60, 63]. Moreover, TMB did not correlate with the proportion of CD8 T cells estimated by CIBERSORT using gene expression profiles [60]. These studies also demonstrated that the combination of gene expression profile-based biomarkers and TMB could improve the prediction of response to ICB therapy [60, 61]. Consistently, our EIC score had no significant correlation with TMB, suggesting that the combination of the EIC score and TMB may improve the prediction of response to immunotherapy. Specifically, the patients who had high EIC scores and low TMB may have the higher likelihood of immunotherapy resistance, but this needs to be validated in patients treated with immune checkpoint inhibitors in clinical trials. Additionally, the genomic differences (mutation burden and neoantigen) of tumour between the EIC and the rest class in LUSC were also similar to those in other tumours such as head and neck squamous cell carcinoma [66] and hepatocellular carcinoma [67]. These evidences suggested that tumour-intrinsic mutations may not impact immunosuppressive microenvironment and the EIC score is independently predictive for potential immunotherapy resistance. However, we found that the EIC had a lower chromosomal alteration burden and a lower frequency of copy number alteration for the driver genes (e.g., *AKT1*, *DDR2*, *KEAP1*), suggesting that copy number alteration may play an important role in regulating immunosuppressive microenvironment.

The robustness of EIC was successfully verified in three validating datasets including 374 LUSC samples. The EIC in the validation datasets also showed potential resistance to ICB therapy and tended to poor prognosis, verifying its predictive value. However, this finding needs further validation on LUSC patients treated with ICB therapy.

Understanding the molecular features of immunosuppressive TME is critical for finding successful solutions of TEX reversion and immunotherapy. Comprehensively multidimensional data analysis unfolded a complex immunosuppressive network that may be dominated by cytokines in TME for LUSC. In our study, tumours within the EIC overexpressed PD-1 and CTLA-4, but did not have a higher tumour mutation burden than those within the rest class. However, recent clinical trials indicated that patients benefitting from anti-PD-1 antibody plus anti-CTLA-4 antibody were associated with a high tumour mutational burden. Therefore, patients within the EIC may not be able to respond to the immunotherapy with the two antibodies. Our comparison between the EIC and the rest class indicated that signal alterations related to TGF-β occurred in both transcriptome and epigenome. Thus, patients within the EIC may benefit from TGF-β inhibition plus ICB. In this regard, a phase 1b/2 clinical trial testing the novel TGF-ß inhibitor, galunisertib, in combination with nivolumab in advanced refractory solid tumours and in recurrent or refractory NSCLC, or in hepatocellular carcinoma, is currently ongoing (NCT02423343). The rest patients who was lacking of the characteristics of immunosuppressive TME may be selected by immunohistochemical assay to determine the tumour-expressed PD-L1 status for eligibility.

Protein expression analysis indicated that PD-L1 and IDO were significantly increased in EIC, and this was consistent with the high transcriptomic expressions of two corresponding genes. IDO is an immune regulatory enzyme which suppresses T cell response. A recent study demonstrated that high IDO activity is associated with primary resistance to immunotherapy in NSCLC [64]. In our study, a high IDO protein expression level illustrated the potential immunotherapy resistance of EIC. Volaric’s study based on immunohistochemistry also demonstrated that IDO is a targetable mechanism of immune resistance frequently coexpressed with PD-L1 [75]. In addition, through checking immunohistochemically stained tissue images from HPA, we found that LUSC patients had different IDO protein expressions. Overall, these evidences suggested that IDO immunohistochemistry may be a biomarker for identifying patients with potential immunotherapy resistance.

## Conclusions

In conclusion, we identified an immunosuppressive class accounting for approximately 30% LUSC patients, which had elevated PD-L1 expression but showed potential resistance to ICB therapy and uniquely immunosuppressive molecular features of TME. Our findings provide new insights for understanding the molecular mechanism of ICB therapy resistance and tailoring appropriate immunotherapy strategies for patients with different molecular characteristics.

## Supplementary Information

**Additional file 1: Figure S1**. Analysis flowchart of this study. **Figure S2**. Identification and characterization of the exhausted immune expression pattern. **Figure S3**. The external validation of the Exhausted Immune class.

**Additional file 2: Table S1**. Publicly available data sets used in this study. **Table S2**. Tools and software used in this study. **Table S3**. Publicly available gene signatures used in the study. **Table S4**. Exhausted Immune classifier (167 genes) with significantly different expression between the Exhausted Immune class and the rest class. **Table S5**. Differently methylation comparison of the 216 CpG located in the promoters of 162 exhaustion related genes. **Table S6**. Correlation between gene expression and methylation in the 162 exhaustion related genes with differentially methylated promoter.

## Data Availability

All data analysed during this study were retrieved from public repositories, which have been listed in the Additional files. The source codes generated to build the web application are available at the GitHub repository (https://github.com/Lilab-SYSU/LUSC_Tex) [43].

## Abbreviations

*BTLA*: Band T lymphocyte attenuator
*CCR4*: C-C Motif Chemokine Receptor 4
*CTLA-4*: Cytotoxic T lymphocyte antigen-4
EIC: Exhausted immune class
GEO: Gene Expression Omnibus
GSEA: Gene set enrichment analysis
HCC: Hepatocellular carcinoma
HPA: Human Protein Atlas
ICB: Immune checkpoint blockade
IDO: Indoleamine 2,3-dioxygenase
IFNγ: Interferon gamma
*IRF7*: Interferon regulatory factor 7
*LAG3*: Lymphocyte activation gene 3
LUAD: Lung adenocarcinoma
LUSC: Lung squamous cell carcinoma
*MYO1G*: Plasma membrane-associated class I myosin
NMF: Non-negative matrix factorization
NK: Natural killer
NSCLC: Non-small-cell lung cancer
OS: Overall survival
*PD-1*: Programmed death 1
PD-L1: Programmed Cell Death 1 Ligand 1
PFS: Progression-free survival
RNAseq: RNA sequencing
ROC: Receiver operating characteristic
RPPAs: Reverse-phase protein arrays
ssGSEA: Single sample gene set enrichment analysis
TCGA: The Cancer Genome Atlas
TIDE: The tumour immune dysfunction and exclusion
TEX: T cell exhaustion
TGF-β: Transforming growth factor-beta
*TIGIT*: T cell immunoreceptor with Ig and ITIM domains
TILs: Tumour-infiltrating lymphocytes
*TIM-3*: T cell immunoglobulin and mucin domain protein
TKIs: Tyrosine kinase inhibitor
TLS: Tertiary lymphoid structure
TMB: Tumour mutational burden
TME: Tumour microenvironment
