# Supplementary material for: Identification of a cytokine-dominated immunosuppressive class in squamous cell lung carcinoma with implications for immunotherapy resistance
Source: Genome Med. 2022 Jul 8;14:72. doi: 10.1186/s13073-022-01079-x (PMC9264601; doi:10.1186/s13073-022-01079-x)

Supplementary Figures

Fig S1. Analysis flowchart of this study. A total of 624 LUSC samples were analyzed in this study. 250 late-stage TCGA LUSC were virtually micro-dissected to identify the Exhausted Immune class. 247 early-stage TCGA LUSC were used for internal validation. Two independent public datasets were used for external validation.

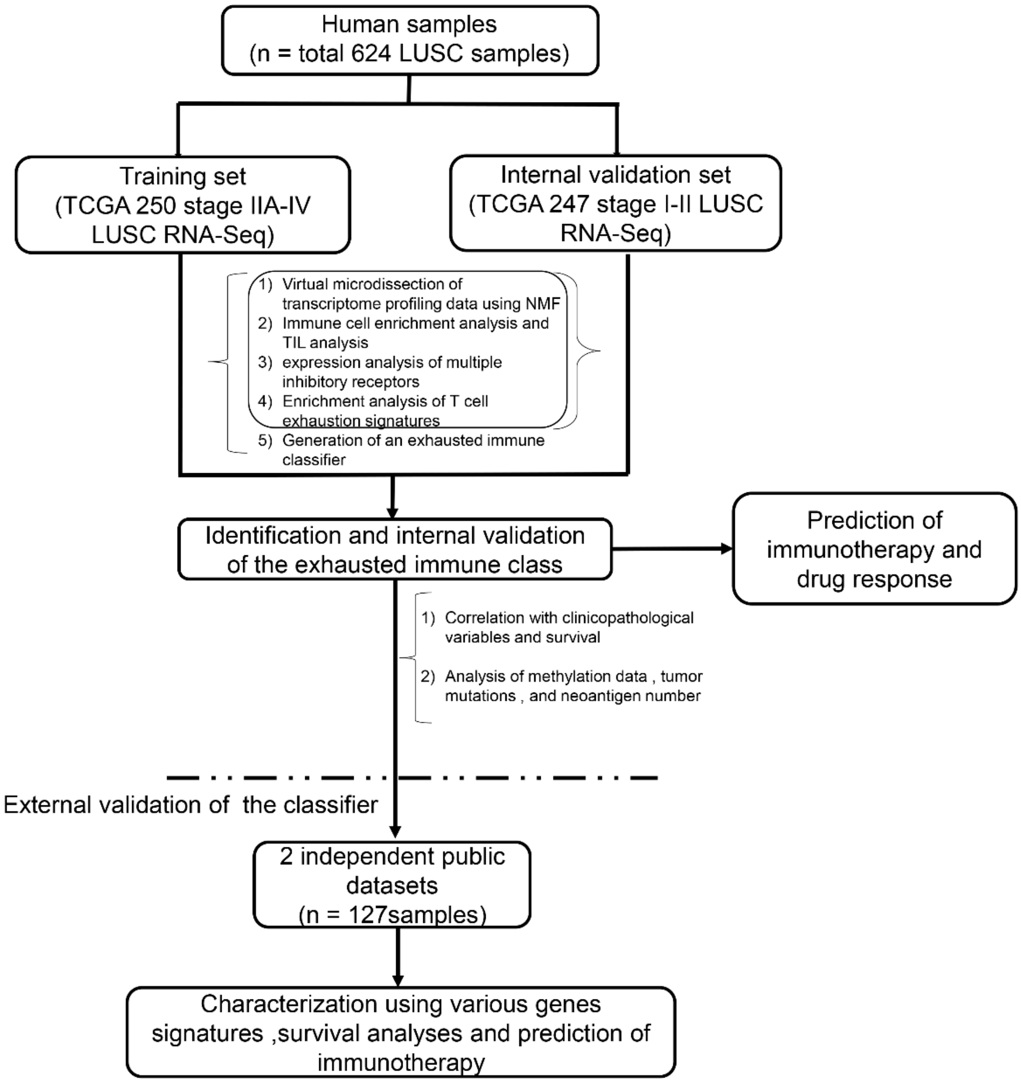

Fig S2. Identification and characterization of the exhausted immune expression pattern. (A) Estimation of the factorization rank for non-negative Matrix Factorization (NMF, r=4 factors, R package) to dissociate distinct expression patterns from RNA-Seq data of 250 TCGA LUSC samples. (B & C) Pathway and hallmark characterization of the Exhausted Immune class. Gene set enrichment analysis (GSEA) between the Exhausted Immune class (n=91) and the rest patients of the cohort (n=169) confirmed the enrichment of inflammation-related pathways, signatures of immune cells, interferon (IFN) signaling and IFN-related signaling pathways (FDR<0.01). (D) Detailed analysis examples of disease-, immunocyte-, and cytokine-related pathways and hallmarks.

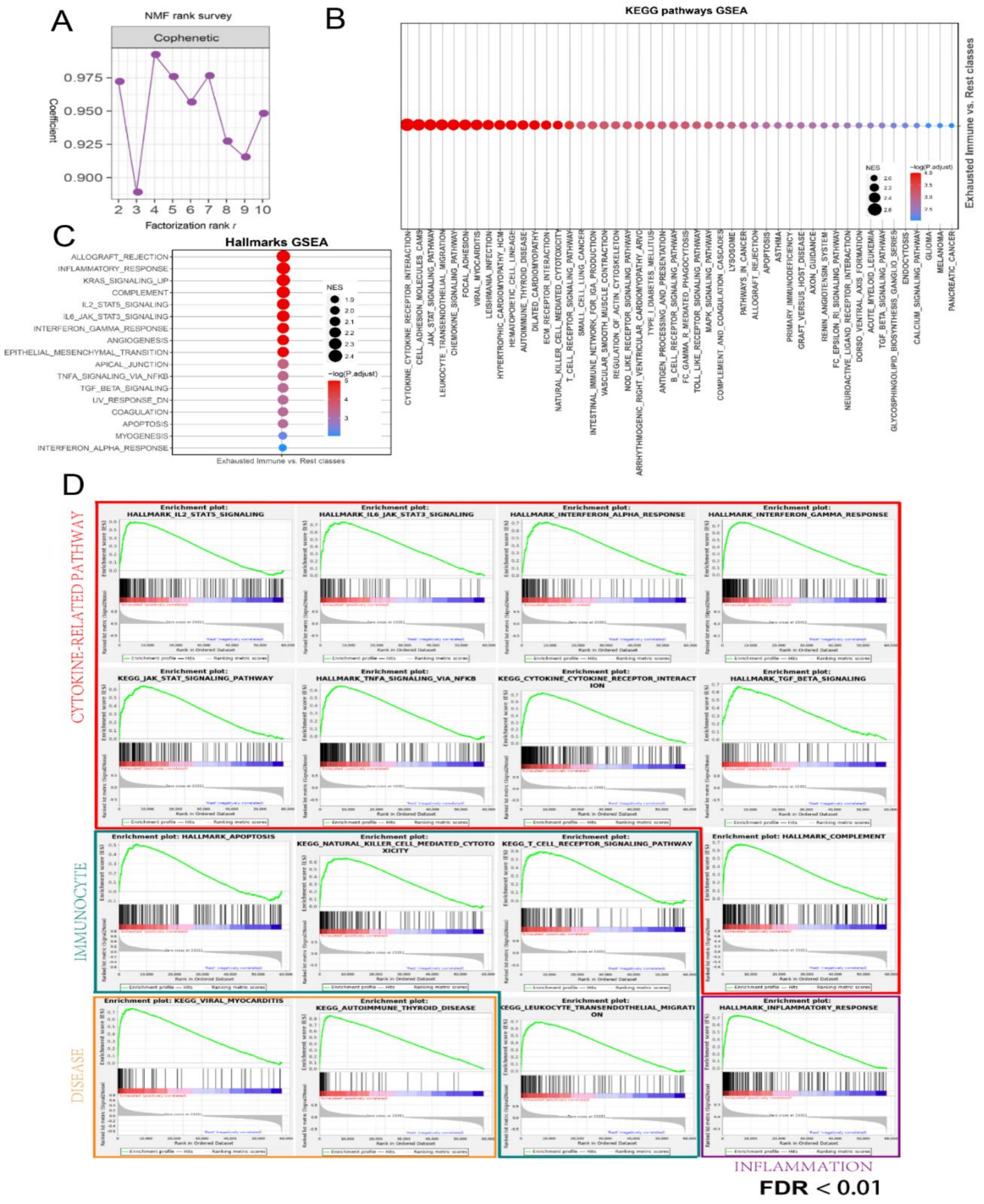

Fig S3. The external validation of the Exhausted Immune class. (A) We performed an unsupervised random forest clustering to cluster 250 TCGA training cohort using 167 Exhausted Immune classifier genes as indicated in the multidimensional scaling (MDS) plot. Blue, exhausted immune group; yellow, rest molecular group. (B & C) Presence and molecular characteristics of the Exhausted Immune class were validated in two publicly independent datasets. (D & E) Kaplan–Meier survival analysis based on the Exhausted Immune class. Patients of the Exhausted Immune class in the two datasets tended to poor prognosis. (F & G) Prediction of response to ICB therapy by TIDE algorithm for the two datasets.

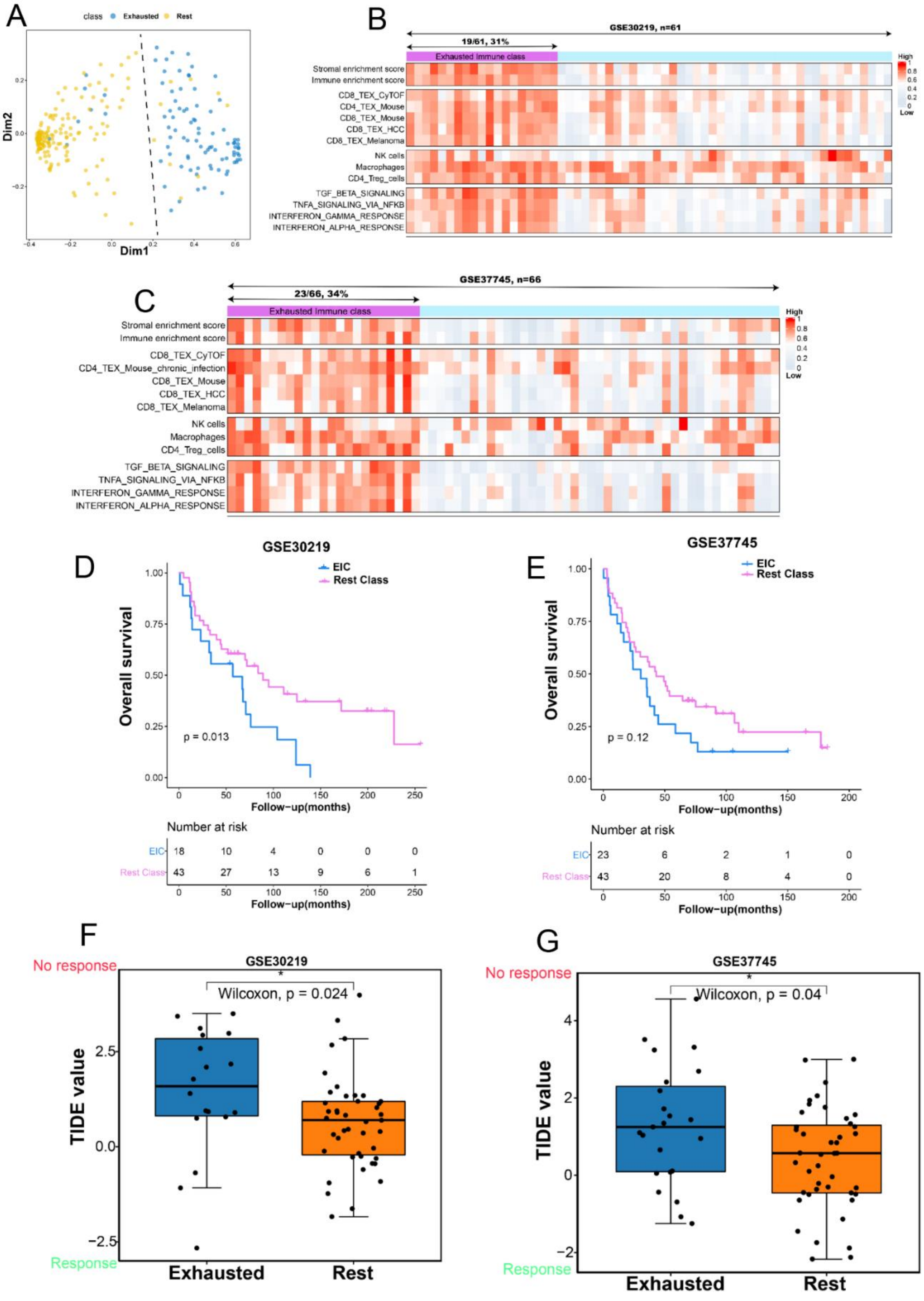

Supplement: Supplementary file 1 — Additional file 1: Figure S1. Analysis flowchart of this study. Figure S2. Identification and characterization of the exhausted immune expression pattern. Figure S3. The external validation of the Exhausted Immune class. [file 13073_2022_1079_MOESM1_ESM.pdf]
